# Establishment of a Transgenic Zebrafish Expressing GFP in the Skeletal Muscle as an Ornamental Fish

**DOI:** 10.31661/gmj.v8i0.1068

**Published:** 2019-01-25

**Authors:** Mohammad Rezaei, Mohsen Basiri, Seyedeh-Nafiseh Hasani, Behrouz Asgari, Hadis Kashiri, Ali Shabani, Hossein Baharvand

**Affiliations:** ^1^Fishery Faculty, Gorgan University of agriculture science and natural resources, Gorgan, Iran; ^2^Department of Stem Cells and Developmental Biology, Cell Science Research Center, Royan Institute for Stem Cell Biology and Technology, ACECR, Tehran, Iran; ^3^Department of Developmental Biology, University of Science and Culture, Tehran, Iran

**Keywords:** Myosin, Transposase, Injection, Plasmid

## Abstract

**Background:**

Transgenic animals have a critical role in the advancement of our knowledge in different fields of life sciences. Along with recent advances in genome engineering technologies, a wide spectrum of techniques have been applied to produce transgenic animals. Tol2 transposase method is one of the most popular approaches that were used to generate transgenic animals. The current study was set out to produce an ornamental fish, which express enhanced green fluorescent protein (EGFP) under control of mylpfa promoter by using Tol2 transposase method.

**Materials and Methods:**

Polymerase chain reaction (PCR) cloning method was performed to insert zebrafish myosin promoter (mylpfa) into Tol2-EGFP plasmid at the upstream of EGFP. In vitro transcription method was used to prepare the transposase mRNA. The Tol2-EGFP plasmid and transposase mRNA were then co-injected into the one-cell stage of zebrafish zygotes. After two days, the fluorescent microscopic analysis was used to select transgenic zebrafishes.

**Results:**

Our data showed that the optimum concentration for recombinant Tol2 vector and transposase mRNA were 50 ng/ul and 100 ng/ul, respectively. The results also revealed that the quality of embryos and quantity of injected construct had the important effects on Tol2 transposase method efficiency.

**Conclusion:**

Data showed that Tol2 transposase is an appropriate method to generate zebrafish transgene. Our finding also showed that mylpfa promoter is a strong promoter that can be used as a selected promoter in the ornamental fish industry.

## Introduction

Generation of first genetically modified bacteria by Cohen and Boyer in the 1970s is perhaps one of the outstanding events in the genetic engineering area [1]. This model was acted as a starting point of establishment transgenic organism technology, and it opened up a new promising avenue for creation a wide range of transgenic animal models. Transgenic technology has also played significant roles in economic and scientific progress. Nowadays, the utilization of transgenic animals is very common in the pharmaceutical, biotechnological and agricultural industries [2-4].

Along with recent advances in genome engineering technologies, various techniques have been developed to produce transgenic animals. Tol2 transposase method is one of the most popular approach that was used to generate transgenic animals [5-7]. In 1996, Koga *et al*. reported the novel transposable elements in the genome of the medaka fish [8]. They called this transposable element Tol2 and showed that the multiple copies of Tol2 elements present in the genome[8]. Other studies revealed that unlike the other transposons, which present in the vertebrate genomes, Tol2 elements are autonomously active [9]. Further, several studies showed that the Tol2 transposase system is a beneficial tool for a wide range of investigations in the field of genome manipulation such as gene transfer and transgenic technology [5, 6]. Transgenic fish (genetically modified fish) trade plays a critical role in the economic growth of some countries [10, 11]. According to Food and Agriculture Organization (FAO) of the United Nations’ report in 2014, about 150 billion dollars has been invested in the fishery products [12], which a considerable amount of this cost invested in the production of transgenic products, such as transgenic fish [12]. Transgenic fish can be used in scientific research as an animal model [4, 13, 14]. Also, it is worth noting that the ornamental fish keeping is one of the most important hobbies in many countries. European countries are the largest consumer market for ornamental fish [11, 15]. Therefore, another potential and interesting application of transgenic fish is keeping them as a pet. The zebrafish (*Daniorerio*) is a tropical freshwater fish, and it has some unique features that attracted the attention of researchers. These features include the genetic similarity to humans, the production of large number offspring, the transparency at early developmental stages, the easy husbandry and care, the potential of regenerative capacity in most organs, and the extremely fast development [13, 16]. The aim of this study was to produce and establish Tg (mylpfa:egfp) zebrafish utilizing Tol2 transposase method. The mylpfa promoter was chosen to drive enhanced green fluorescent protein (EGFP) expression in the muscle cells. As an animal model, transgenic zebrafish has enormous potential in various applications in research and industry.

## Materials and Methods

### Zebrafish Maintenance

Wild-type TU strain was maintained under standard conditions (at 28 °C on a 14-h light/10-h dark cycle) in the aquatic automated maintenance system. Zebrafish were fed on a commercial diet twice a day. All animal protocols were approved by the Institutional Animal Care and Use Committee of Royan Institute. (Approval code: IR.ACECR.ROYAN.REC.1394.76)

### Material Preparation

Tol2-EGFP vector was used as baseline vector, and Platinum® Taq DNA Polymerase High Fidelity was purchased from Invitrogen Company (San Diego, CA, USA). Other reagents, including Plasmid extraction kit, DNA gel extraction kit, were purchased from MACHEREY-NAGEL Company (Germany).

### Genomic DNA Extraction

Genomic DNA was extracted from fin samples of the adult zebrafish using a DNA extraction Kit, according to the manufacturer’s instructions (Invitrogen, USA, Cat No: 13323).

### Designing and Cloning Construct

Polymerase chain reaction (PCR) cloning method was used in order to insert zebrafish myosin promoter (gene ID: 30429, mylpfa) into Tol2-EGFP plasmid at the upstream of EGFP. Primers were then designed to amplify 2000 bp upstream of the mylpfa coding sequence (CDS), and SphI and SalI restriction sites were flanked to 5ʹof forward (CAACGCATGCAGAGGAATGAGCCACCAACTC) and reverse (TATAGTCGACACGGTATGTGTGAAGTCTAAG) primers, respectively. NCBI primer-BLAST (https://www.ncbi.nlm.nih.gov/tools/primer-blast/) was used as primers designing tool. The purified PCR product by Gel Extraction Kit (MACHEREY-NAGEL, Germany) was ligated into Tol2-EGFP cloning vector. The recombinant vectors were transformed into competent E. coli Top10. Ampicillin resistance was used in order to screen the bacterial colonies harboring recombinant plasmid DNA. Then colony PCR using M13F (5’-TGTAAAACGACGGCCAGT-3’) and M13R (5’- CAGGAAACAGCTATGACC-3’) primers were performed to investigate recombinant vector. The colony PCR product was visualized by 1% agarose gel electrophoresis. The recombinant plasmid colonies were then selected and purified by Plasmid Miniprep Kit (MACHEREY-NAGEL, Germany) according to the manufacturer’s instructions. Recombinant plasmids were finally confirmed by digestion and sequencing methods, respectively.

### Transposase in Vitro Transcription (IVT)

At first step of IVT, the pCS˗transposase plasmid was linearized with NotI restriction enzyme and followed by ethanol precipitation to remove proteins that may interfere IVT reaction. mMESSAGE mMACHINE SP6 Transcription Kit (#cat AM1340) was used for transposase in vitro transcription. According to the kit’s instruction, following components of IVT including 4 µl nuclease-free water, 2µl 10X buffer, 10µl 2X NTP/CAP, 2 µl or 1µg linearized plasmid, and 2 µl sp6 enzyme were added to the reaction tube respectively and incubated at 37°C 3 hrs. Then, DNase treatment was performed to eliminate the plasmid DNA contamination followed by Lithium Chloride (LiCl) precipitation to remove unincorporated nucleotides and proteins.

### Microinjection

The recombinant plasmid and transposase mRNA were co-injected into the one-cell stage zebrafish zygotes with three different concentrations (Low, medium and high) in order to find optimum concentrations. Injections volume in this study was 1nL. The injected embryos were evaluated at 2 days post fertilization (dpf) using fluorescence stereo-microscope in order to select the embryos that express EGFP in their muscles. EGFP positive embryos were then chosen and raised as F0 generation of Tg(mylpfa:egfp) zebrafish. Each one of the F0 generation adult zebrafish was crossed with a wild-type (TU stain) zebrafish to identify founder and produce F1 generation.

## Results

**Figure 1:**
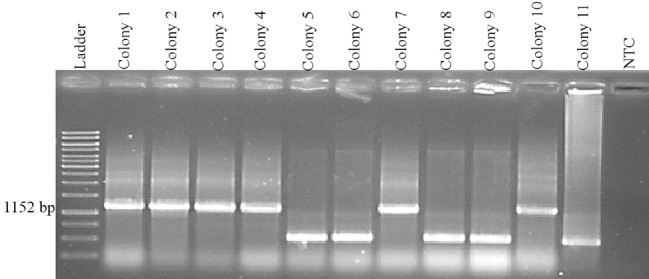
Colony PCR using M13 primers. PCR product size 1152 bp indicated the positive colonies.

The mylpfa promoter was amplified by PCR. PCR product was then cloned into Tol2-EGFP vector [17]. In the next step, colony PCR was used to determine the recombinant constructs (Figure-1). Six colonies (1, 2, 3, 4, 7 and 10) were marked as the positive colonies according to colony PCR results (1152bp colony PCR product). The recombinant plasmids were finally confirmed by sequencing method (Pishgam company, Iran), using M13 forward and reverse universal primers. The sequencing results were subsequently examined by CLC Sequence Viewer 5 software (QIAGEN Bioinformatic).

### IVT

**Figure 2:**
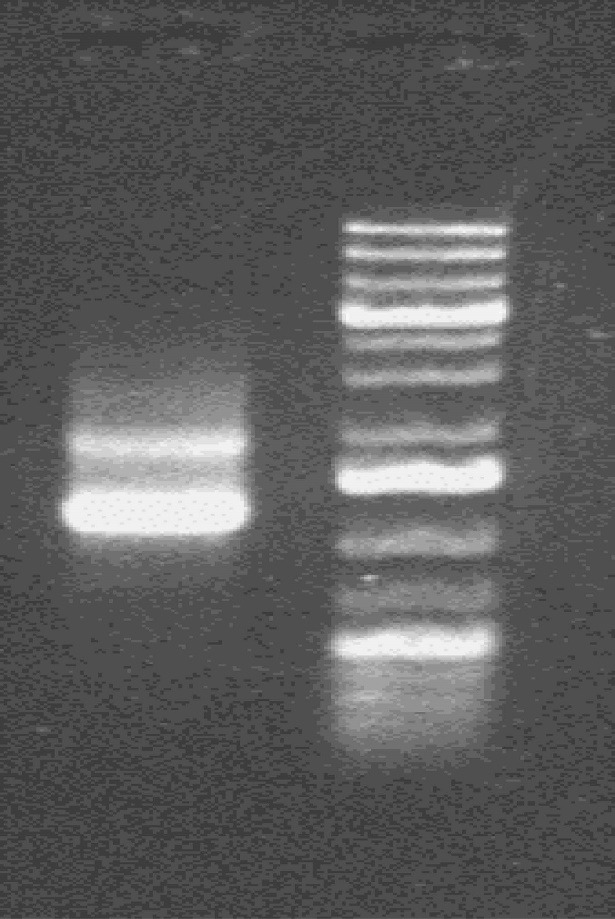
Transposon mRNA after DNase treatment (ladder 1kb Plus)

The ITV was performed according to the manufacturer’s instruction to make transposase mRNA. To observe the results, 2µl of the mRNA product was mixed with 2µl Orange G loading buffer and 1 % agarose gel electrophoresis was then carried out(Figure-2).

### Microinjection

The recombinant plasmid and transposase mRNA were co-injected into the one-cell stage of zebrafish embryos with low (10 ng/ul and 50 ng/ul), moderate (50 ng/ul and 100 ng/ul) and high (100 ng/ul and 150 ng/ul) concentrations, respectively. The results showed that the high concentration of constructs had a toxic effect on embryos. Our results also revealed that the optimum concentration of recombinant Tol2 plasmid and transposase mRNA were50 ng/ul and 100 ng/ul, respectively (Table-1).

### Mosaic Expression

**Figure 3:**
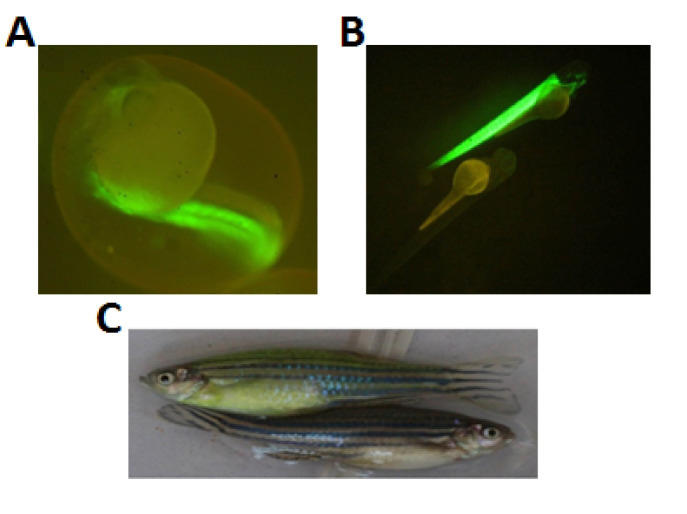
Microscopic evaluation of embryos. **A:** Two dpf embryos. **B:** Five dpf embryos. **C:** Adult wild-type and Tg(mylpfa:egfg) zebrafish.

**Figure 4:**
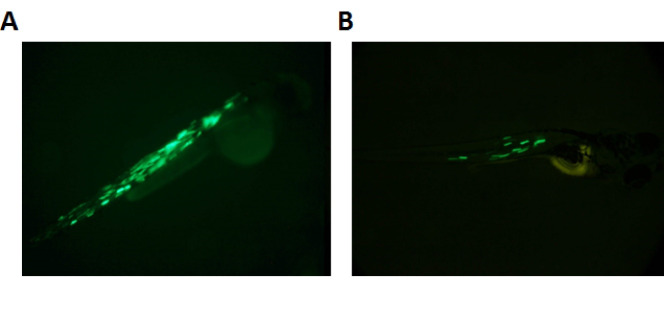
Mosaic expression. **A:** High expression. **B:** Low expression.

The recombinant plasmid and transposase mRNA were co-injected into 142 one-cell stage of zebrafish embryos. Of all, 18 were marked EGFP positive at 2 dpf using fluorescence stereo-microscope evaluation (Figure-3). We observed different mosaic expression between the transgenic zebrafishes that could be related to the different insertion time of DNA plasmid into the fish genome (Figure-4). The high expression shows that DNA inserted in the early stage of development so they could be more prone for germinal transmission. We raised all of the transgenic zebrafishes until adult size (about 3 months). With growing up, green color became mosaically visible on some of the fishes.

### Germinal Transmission

Each one of the F0 generation adult zebrafish (18 EGFP positive embryos) was crossed with a wild-type (TU strain) zebrafish for germinal transmission assay. We take at least 100 live embryos from any fish as germinal transmission could be variable (between 3-100%). Microscopic evaluation was performed to detect EGFP positive F1 embryos. From 18 EGFP positive F0 fishes, one of them showed germinal transmission and consequently considered as founder fish and F1 generation of Tg(mylpfa:egfp) zebrafish (Figure-3). The founder fish was female and germinal transmission efficiency was about 38% (120/321 embryo). The green color of our founder fish was more visible than others; so the F0 fishes with higher expression have more chance for germinal transmission.

## Discussion

In this study, we used the Tol2 transposase method to produce the transgenic zebrafish, because previous studies showed the simplicity and high efficiency of this method for fish transgenesis[5, 18, 19]. We selected mylpfa promoter to drive EGFP expression in muscle tissues. The efficiency of Tol2 transposase method was very low in this research (12.5%), while the efficacy of this method that reported by other studies were more than 50 percent [20, 21]. It seems that the quality of embryos and quantity of injected construct can be the reason for the low efficiency of Tol2 transposase method in our study[22]. Our transgenic zebrafishes expressed the high intensity of EGFP, which could be seen with the naked eyes. The explanation of this observation is the strong activity of the mylpfa promoter. It is also important to note that the majority of the body mass consists of the muscle tissue.

The strong activity of the mylpfa promoter is in accordance with the previously reported study by Gong *et al*. [23]. They demonstrated the intense activity of mylpfa promoter, and the size of muscle mass makes the color of the fluorescent protein visible to the naked eye. Regarding fish, muscle cells have a high capacity of recombinant proteins expression. These authors suggested that mylpfa transgenic zebrafish can be used as a new transgenic bioreactor system [23]. Moreover, it has been shown that Tg (mylpfa:egfp) zebrafish can be used as a biosensor for the detection of organophosphorus pollutants in the water. In 2014, Almeida *et al*. published a paper in which they described that treatment of Tg (mylz2:DsRED) zebrafish by methyl parathion, as an organophosphate compound, can downregulate the expression of DsRED fluorescent protein in this animal model [24].Furthermore, in an elegant study in 2010, Gabillard *et al*. demonstrated that heat stress could affect muscle cell differentiation by using MLC2f-gfp transgenic trout that exhibited fluorescence in their muscle tissues[25]. Another potential and interesting application of the Tg (mylpfa:egfp) zebrafish is its use as an ornamental fish. The ornamental fish trade is a global industry that has great potential to generate jobs in every society [11, 15]. Color plays a critical role in ornamental fish trade [23], and it is useful to remind that strong activity of mylpfa promoter and the size of muscle mass are two important factors that can be used to produce a different color of ornamental fishes. It is necessary to mention that the high expression of recombinant fluorescent protein did not have any adverse effects on viability or normal activity of the transgenic zebrafishes.

## Conclusion

In the present study, Tol2 transposase method was used to produce Tg(mylpfa:egfp) zebrafish. Our data showed that Tol2 transposase is an appropriate method to generate zebrafish transgene and showed no any negative effect on viability or normal activity of the transgenic zebrafish. Genome manipulation methods open a new avenue for study and evaluation of cellular and molecular events in normal and pathological conditions. In this study, EGFP expression in muscle cells provides an opportunity for monitoring of cellular and physiological activities in any desired conditions. Our finding also showed that mylpfa promoter is a strong promoter that can be used as a selected promoter in the ornamental fish industry.

## Acknowledgment

This work was supported by Royan Institute and Gorgan University of agriculture science and natural resources. (grant number: 94000126)

## Conflict of Interest

The authors declare that there is no conflict of interest.

**Table 1:** Different Concentrations of Constructs Micro Injection

**Concentration**	**Total**	**Dead**	**Live**	**GFP Positive**
**Low**	182	16	166	0
**Moderate**	142	41	101	18
**High**	134	131	3	0
